# To stitch or not to stitch: the skin closure of laparoscopic port sites, a meta-analysis

**DOI:** 10.1007/s00464-022-09269-9

**Published:** 2022-05-24

**Authors:** Lucy P. Aitchison, Andy Z. L. Chen, Clare Toms, Charbel Sandroussi, David A. Yeo, Daniel Steffens

**Affiliations:** 1 Surgical Outcomes Research Centre (SOuRCe), The University of Sydney and Sydney Local Health District, Sydney, NSW Australia; 2 Faculty of Medicine, Prince of Wales Clinical School, The University of New South Wales, Sydney, NSW Australia; 3 Department of Surgery, Royal North Shore Hospital, St Leonards, Sydney, NSW 2062 Australia; 4 Department of Surgery, Westmead Hospital, Sydney, NSW Australia; 5 RPA Institute of Academic Surgery (IAS), Royal Prince Alfred Hospital and University of Sydney, Sydney, NSW Australia; 6 Faculty of Medicine and Health, Central Clinical School, The University of Sydney, Sydney, NSW Australia; 7 Department of Upper Gastrointestinal and Hepatobiliary Surgery, Royal Prince Alfred Hospital, Sydney, NSW Australia

**Keywords:** Laparoscopic surgery, Skin closure, Tissue adhesive, Suture, Surgical staples

## Abstract

**Background:**

Previous meta-analyses examining skin closure methods for all surgical wounds have found suture to have significantly decreased rates of wound dehiscence compared to tissue adhesive; however, this was not specific to laparoscopic wounds alone.

This study aims to determine the best method of skin closure in patients undergoing laparoscopic abdominopelvic surgery in order to minimize wound complications and pain, while maximize cosmesis, time and cost efficiency.

**Methods:**

A comprehensive search of EMBASE, Medline, Pubmed, and CENTRAL was conducted from inception to 1st May 2020 for randomized controlled trials (RCTs). Two independent reviewers extracted data and assessed risk of bias. The Grading of Recommendations Assessment, Development and Evaluation (GRADE) system was used to describe the quality of evidence. Meta-analysis was performed using a random-effects model. A summary relative risk (RR) was calculated for dichotomous outcomes where data could be pooled. (Prospero registration number: CRD42019122639).

**Results:**

The literature search identified 11,628 potentially eligible studies. Twelve RCTs met inclusion criteria. There was no difference in wound complications (infection, dehiscence, and drainage) between sutures, tissue adhesives nor adhesive papertape. Low-quality evidence found transcutaneous suture had lower rates of wound complications compared with subcuticular sutures (RR 0.22, 95%: CI 0.05–0.98). There was no evidence of a difference in patient-evaluated cosmesis, prolonged pain, or patient satisfaction between the three groups. Closure with tissue adhesive and adhesive papertape was faster and cheaper than suture.

**Conclusion:**

Tissue adhesive and adhesive papertape offer safe, cost and time-saving alternatives to closure of laparoscopic port sites compared to suture.

**Supplementary Information:**

The online version contains supplementary material available at 10.1007/s00464-022-09269-9.

Laparoscopic surgery has become the standard of care for surgical procedures across multiple specialties, reducing perioperative complications, accelerating recovery and providing superior cosmetic results [1]. The skin closure method should aim to keep the skin closely opposed during the hemostatic and inflammatory healing phases until the overlapping proliferative phase is able to provide tensile strength [2]. Excess trauma and foreign material have the potential to prolong the inflammatory phase. Prolonged inflammation is detrimental to the healing process and increases pain, worsens cosmesis and increases infection risk [3].

Skin closure of laparoscopic port sites can be achieved with suture, tissue adhesive, adhesive papertape or staples. Globally, material and technique vary widely, and is largely dependent on training exposure and local opinion. There is currently no consensus as to the optimal method of closure of the skin following laparoscopic surgery.

Tissue adhesives (most commonly 2-octyl-cyanoacylate and n-2-butyl-cyanoacrylate) are liquid monomers that undergo an exothermic reaction upon exposure to a moist surface, polymerizing to provide a strong tissue bond [4]. Adhesive papertape for skin closure is made of a porous, non-woven material that is reinforced with polyester filaments for strength and coated with adhesive and iodophor. Both tissue adhesives and adhesive papertape do not leave hatch marks and do not require follow-up for removal (unlike transcutaneous sutures). It is hypothesized that tissue adhesive and adhesive papertape are quicker to apply, induce less of a foreign body reaction than suture or staple, and thus may have improved cosmesis and pain, with no increased complication rate.

To our knowledge there are currently two meta-analyses comparing methods of skin closure in laparoscopic surgery. Sajid et al. compared tissue adhesive with suture and included four RCTs [5]. However, their study is out of date, limited their methods to tissue adhesive and suture only, and did not consider strength of evidence, for example using the Grade of Recommendations Assessment, Development, and Evaluation (GRADE) system [6]. A Cochrane meta-analysis published in 2014 is more recent. However, it evaluates all wound closures, not only laparoscopic wounds. These broad inclusion criteria are unable to guide clinical decision-making in laparoscopic surgery [7].

Through a meta-analysis of randomized controlled trials, this study aims to determine the best method of skin closure in patients undergoing laparoscopic abdominal or pelvic surgery. The objective of this study is to aid surgeons in making an evidence-based decision on the concluding surgical step of any laparoscopic procedure, to minimize wound complications and pain, and maximize cosmesis, time and cost efficiency.

## Materials and methods

The Preferred Reporting Items for Systematic Reviews and Meta-Analyses (PRISMA) Statement [8] as well as the Cochrane Handbook for systematic reviews of interventions were used to guide the conduct and reporting of this study [9].

### Protocol and registration

PRISMA-P was used as a guide to establish the review protocol prior to commencement. The protocol was registered in Prospero (registration number: CRD42019122639). Webpage: https://www.crd.york.ac.uk/prospero/display_record.php?RecordID=122639.

### Eligibility criteria

Studies: Randomized controlled trials comparing two or more methods of closure of laparoscopic port sites.

Participants: Patients undergoing elective laparoscopic abdominal or pelvic surgery, across all relevant surgical specialties, including general surgery, urology and gynecology, with no age limitation.

Interventions: Laparoscopic port site skin incision closure comparing two or more methods, including but not limited to suture, staple, tissue adhesive and adhesive papertape. Closure of fascia was not examined. Studies were excluded if they compared different materials within the same method of closure (for example, two different types of tissue adhesive).

### Outcomes

Primary outcome: Wound complications including dehiscence, infection (superficial surgical site infection only), persistent erythema or drainage.

### Secondary outcomes

- Cosmetic appearance (evaluated by any validated scale including the Visual Analog Scale (VAS) or Hollander Wound Evaluation Scale (HWES); evaluation by investigator and by patient assessed as separate outcomes)
- Pain (evaluation of ‘tenderness’ excluded from analysis)
- Overall patient satisfaction (encompassing factors such as need for suture or staple removal)
- Surgeon satisfaction (encompassing practicality and ease in performing the closure technique)
- Time taken to achieve skin closure
- Cost effectiveness.

### Information sources

A comprehensive search was performed in the following electronic databases from the earliest record to 1st May 2020: Medline, PubMed, EMBASE and the Cochrane Central Register of Controlled Trials (CENTRAL).

The search terms and strategies for Medline, EMBASE, PubMed and CENTRAL can be found in Supplementary Table 1 in the Appendix.

Two reviewers (LA, AC) independently screened each citation by title and abstract to identify potentially eligible studies. Full texts were obtained and reviewed by two reviewers (LA, AC) to determine if the inclusion criteria were met. Any disagreements about inclusion were resolved by consensus discussion with a third independent reviewer (DS). Studies deemed ineligible were noted and reasons for exclusion recorded. The reference lists of all included studies and relevant systematic reviews and meta-analyses were reviewed for additional relevant articles.

### Data extraction

Two reviewers (LA, AC) independently extracted the data from the included studies. If required, individual study authors were contacted for clarification or missing data. The studies were excluded from quantitative analysis if further clarification could not be obtained.

### Risk of bias (quality) assessment in included studies

The methodological quality of RCTs was assessed using the Cochrane Risk of Bias tool [10] by two reviewers (LA, AC). Discrepancies between review authors were resolved through discussion and consensus with involvement of a third reviewer (CT).

### GRADE assessment

Quality of evidence was assessed using the GRADE approach [9]. Quality of evidence was downgraded from “high” by one level for each factor that was encountered—limitation in study design, inconsistency of results and imprecision. Evidence from single RCTs with less than 300 participants was downgraded for inconsistency and imprecision. Indirectness was not considered in this review because inclusion criteria for this study already allowed for a specific population, outcomes and direct comparisons.

### Assessment of heterogeneity

Clinical and methodological heterogeneity was evaluated via a risk of bias assessment and the table of characteristics of included studies. Statistical heterogeneity was evaluated using the I^2^ statistic. Heterogeneity was considered to be low if I^2^ < 30%, moderate if I^2^ 30–60% and substantial if I^2^ > 60% [9].

When possible, meta-analysis was performed using a random-effects model. A summary relative risk (RR) was calculated for dichotomous outcomes where data could be pooled. For continuous variable, this was presented as mean difference with 95% confidence interval (CI).

Where median and range were provided, these were converted to mean and standard deviation to allow for pooled analysis using the method outlined by Hozo, Djulbegovic and Hozo [11].

For assessment of costs, all currencies were converted to US dollars. This was performed on 1^st^ May 2020 using https://www.xe.com/currencyconverter/.

## Results

### Study selection

**Fig. 1:**
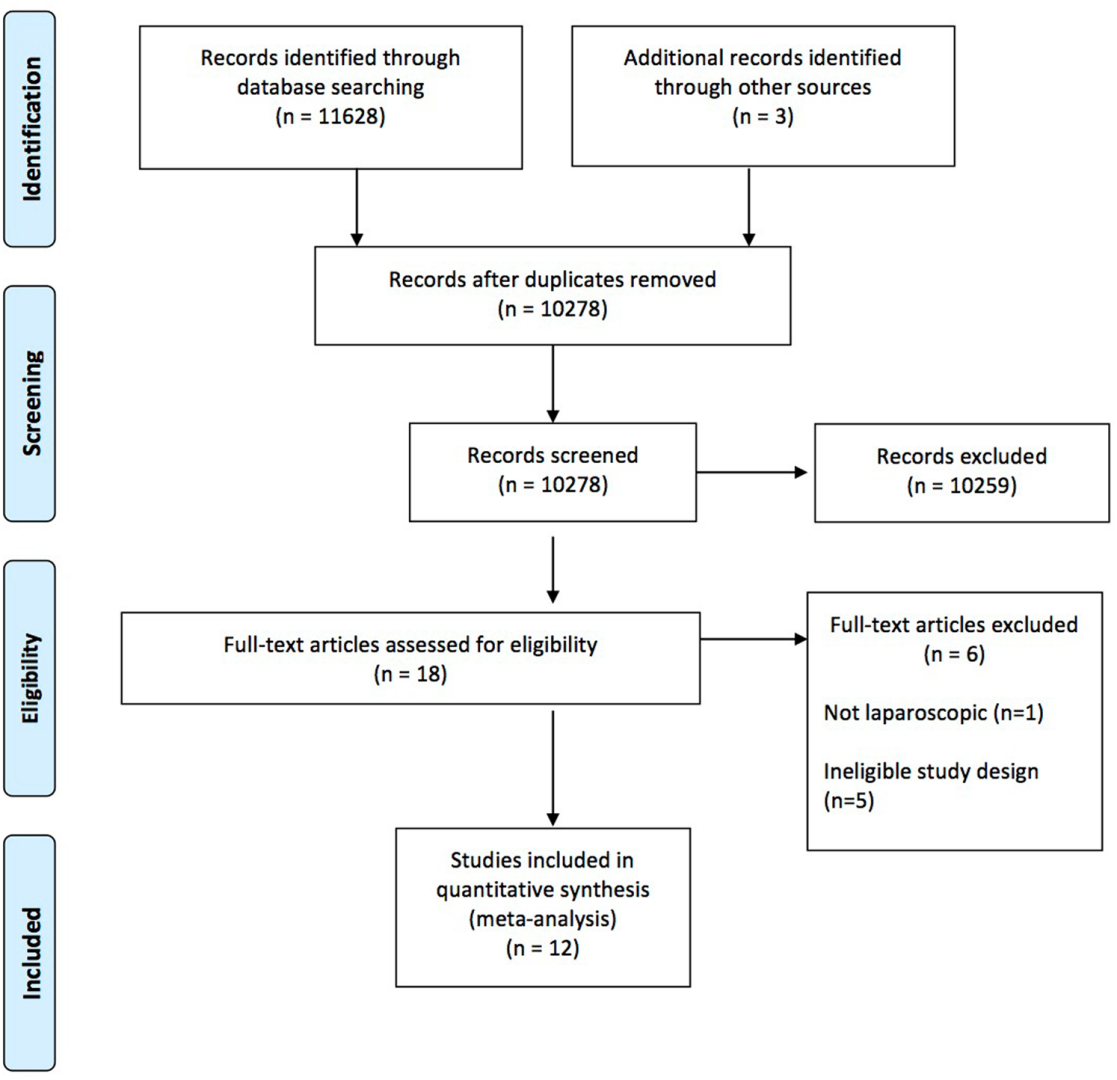
Study flow diagram

The initial electronic medical database searches identified 11,628 potentially eligible studies. After screening title and abstract, a total of 18 full-text studies were assessed for eligibility. Twelve published RCTs met the inclusion criteria and were included in this review. The results of the database searches are summarized in the PRISMA flow diagram (Fig. 1).

### Study characteristics

Twelve studies were included for quantitative analysis with a total of 861 patients. Eleven studies included adults, one reviewed children only [12]. Seven studies were of parallel group design [12–18] and five had split body design (different wounds on the same participant randomized) [19–23]. Nine of the twelve studies compared tissue adhesive with suture [13–18, 21, 22, 24]. Four studies compared adhesive papertape with suture [15, 20, 22, 23]. Three studies compared adhesive papertape with tissue adhesive [15, 20, 22]. None of the included studies used staple as a method of skin closure. Duration of follow-up ranged from 14 to 90 days.

### Risk of bias within studies

**Fig. 2:**
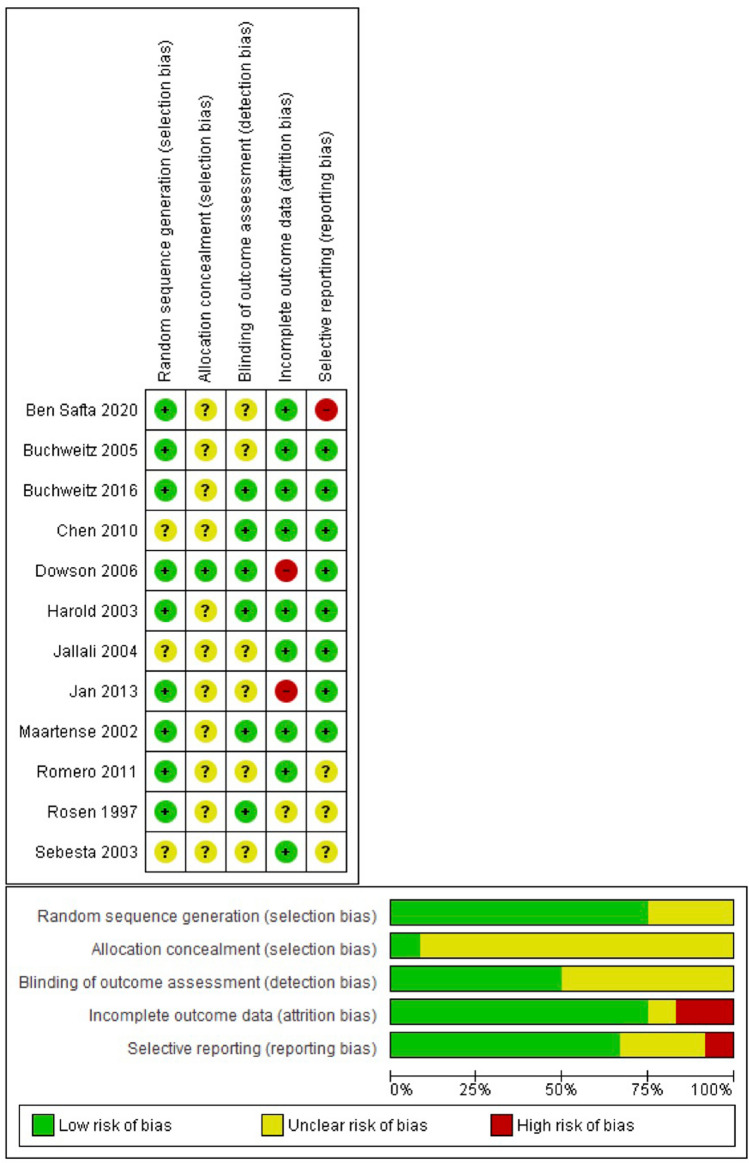
Risk of bias

The results for a quality assessment for included trials are shown in Fig. 2.

Random sequence generation was performed adequately in 10 studies [12, 13, 15–18, 20, 23, 24]. In the remaining studies, the method of randomization was unclear [14, 21]. Given the nature of the intervention it was impossible to blind investigators delivering the intervention, and thus performance bias was unable to be accounted for all studies. Patient blinding is difficult but could theoretically be achieved with the use of opaque dressings. No attempt at this was mentioned in any of the studies. Nine studies had blinded investigators [12, 15, 17, 18, 20–24]. Five studies had outcomes assessed by unblinded patients and are therefore at risk of detection bias [12, 15, 20, 22, 24].

### Comparison 1: suture vs tissue adhesive

#### Primary outcome: wound complications

**Fig. 3:**
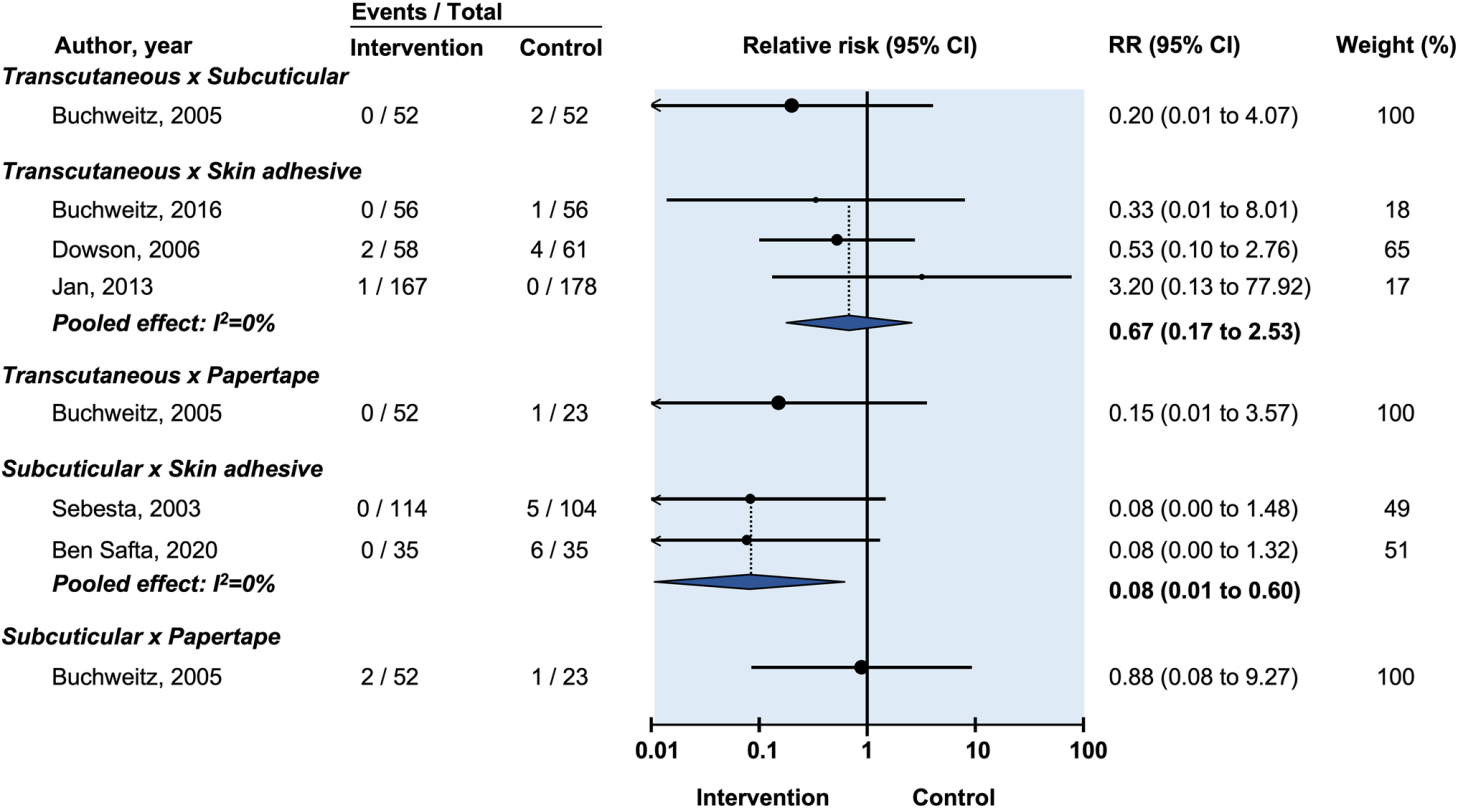
Relative risk of postoperative wound dehiscence in randomized controlled trials on efficacy of optimal wound closure of abdominal laparoscopic port sites. Relative risk (RR) < 1 favor intervention group. CI = Confidence intervals

There was no significant difference in infection risk between skin closure with transcutaneous sutures compared with tissue adhesive (RR: 1.23, 95% CI: 0.48–3.19) (Fig. 3) [18, 24].

Six studies compared subcuticular suture against tissue adhesive for infection [13–15, 17, 21, 22]. However, as four of these studies recorded no cases of infection [12, 17, 21, 22] only data from the remaining two studies contributed to the meta-analysis [14, 15]. There was no evidence that tissue adhesive increases risk of infection compared with skin closure by subcuticular suture (RR: 0.52, 95% CI: 0.15–1.84) (Fig. 3).

Three studies compared transcutaneous suture against tissue adhesive for dehiscence [16, 18, 24]. The pooled effect was not statistically significant (RR: 0.67, 95% CI: 0.17–2.53) (Fig. 3). Data from three studies compared subcuticular suture vs tissue adhesive for dehiscence [13, 14, 17]. The pooled effect was not statistically significant (RR: 0.33, 95% CI: 0.05–5.10) (Fig. 3).

Prolonged erythema lasting more than 14 days was compared between transcutaneous suture and tissue adhesive in two studies [16, 18]. There was no significant difference between the two groups (RR: 1.18, 95% CI: 0.77–1.79) (Fig. 3). Chen et al. reported on prolonged erythema comparing subcuticular suture to tissue adhesive, with a significantly increased rate of persistent erythema at 2–4 weeks follow-up in the subcuticular suture group (RR: 0.06, 95% CI: 0.23–0.45) (Fig. 3) [21].

Two studies compared transcutaneous suture vs tissue adhesive for prolonged drainage lasting more than 14 days [16, 18]. There was no significant difference between the two groups (RR: 0.63, 95% CI: 0.14–3.00) (Fig. 3). Two studies compared subcuticular suture vs tissue adhesive for prolonged drainage [14, 21]. There was no significant difference between the two groups (RR: 3.08, 95% CI: 0.39–24.25).

### Secondary outcomes:

#### Overall patient satisfaction

Dowson et al. found no significant difference between patient satisfaction 24–48 h post operative [18]. Ben Safta et al. also found no difference between patient satisfaction with subcuticular suture and tissue adhesive at 4-week follow-up [13].

#### Surgeon satisfaction

Jan et al. found that 100% of surgeons were satisfied with both suturing and tissue adhesive [16]. Maartense et al. asked surgeons to rate closure methods as very practical, not very practical and not practical. 6.3% found tissue adhesive not practical and 0% found suture not practical (*p *= 0.07) [15].

#### Cosmesis

Three studies compared subcuticular suture with tissue adhesive with VAS by blinded plastic surgeon [15, 17] or by patient [15] at follow-up ranging from 42 to 90 days. Pooling of VAS data was not possible due to inconsistent data reporting; however, all studies found no difference in cosmesis assessed by VAS score between the two groups [15].

**Fig. 4:**
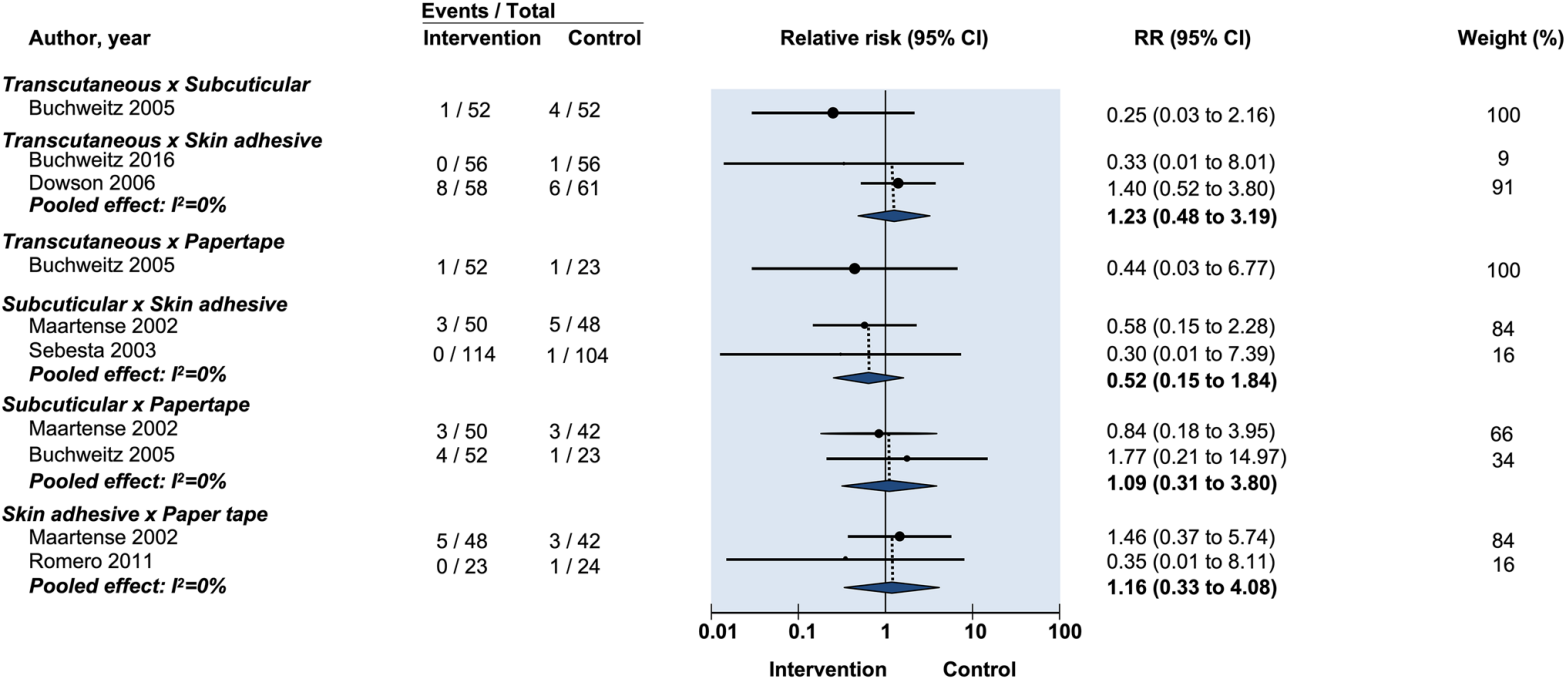
Relative risk of postoperative infection in randomized controlled trials on efficacy of optimal wound closure of abdominal laparoscopic port sites. Relative risk (RR) < 1 favor intervention group. CI = Confidence intervals

Seven studies used HWES to evaluate cosmesis for suture vs tissue adhesive [13, 15–18, 21, 24]. Assessment occurred early [14 days] by Jan et al. [16] and delayed (42–90 days) in the remaining studies. Comparing transcutaneous sutures with tissue adhesive, Dowson et al. reported 100% of their scars rated as a perfect HWES score at 90-day follow-up and could not be included in pooled meta-analysis [18]. Given significant heterogeneity of follow-up times, scores were not pooled for meta-analysis. No study found a significance difference between the two groups (Jan et al. RR: 1.07, 95% CI: 0.99–1.03; Buchweitz et al. RR: 1.02, 95% CI: 0.97–1.07) (Fig. 4) [16, 19].

Four studies compared subcuticular suture with tissue adhesive for cosmesis as assessed by HWES [13, 15, 17, 21], the time frames for follow-up were considered to be too heterogeneous for meaningful pooled analysis (14–28 days [21], 28 days [13], 42–56 days [17] and 90 days [15]). Maartense et al. found significantly higher perfect HWES score as assessed by blinded surgeon in the suture group (97% vs 77%, RR: 1.37, 95% CI: 1.13–1.65) [15]. Ben Safta et al. [13] and Jallali et al. found no significant difference in HWES [17]. Chen et al. found better HWES cosmetic score in the tissue adhesive group at 10–14 days follow-up (5.92 ± 0.05 vs 5.50 ± 0.13, *p *= 0.009) [21].

#### Pain

Buchweitz et al. reported pain using a VAS at 7–12 days and again at 70–98 days whereas the other two studies reported on the presence of pain at 90 days [19]. Therefore data from Buchweitz et al. was not incorporated into pooled meta-analysis. When the two remaining studies comparing transcutaneous sutures vs tissue adhesive were pooled there was no difference in prolonged pain (RR: 1.18, 95% CI: 0.49–2.85) (Fig. 4) [16, 18] (Table 1).

**Table 1:** Characteristics of the included trials (*n* = 12)

Author, year	Study Population	Procedure	Methods of closure			Size of port sites (randomization method)	Outcome measured	Outcome definition
			Group I	Group II	Group III (If applicable)			
Ben Safta (2009)	Sample (% male): 70 (32%); Median age (range): 49 (35–62); BMI: NR	Laparoscopic cholecystectomy	Subcuticular 3–0 poliglecaprone	Tissue adhesive 2- octyl cyanoacrylate	NA	Median (range) 25 mm (20–30) (randomized to same patient)	Complications	ND
							Cosmesis	Patient HWES and POSAS at 1 and 4 weeks; Patient satisfied or not satisfied; VAS
Buchweitz (2016)	Sample (% male): 56 (0%); Mean age (SD): 35.6 (8.81); Mean BMI (SD): 23.20 (3.92)	Gynecological	Transcutaneous suture 3–0 polypropylene	Tissue adhesive 2-octyl and n-butyl cyanoacrylate blend	NA	5 mm (randomized to same patient)	Complications	Wound dehiscence, secretions, redness at 7–12 days and 70–90 days
							Cosmesis	Patient satisfaction with VAS at 70–90 days; Blinded investigator HWES at 70–90 days; Blinded investigator forced choice: which looks better at 7–12 days and 3 months
							Pain	VAS at 7–12 days and 70–90 days
Buchweitz (2005)	Sample (% male): 52 (0%); Mean age (SD): 33.0 (6.70); BMI: NR	Gynecological	Transcutaneous suture 4–0 polyglactin	Subcuticular suture 4–0 polyglactin	Adhesive papertape (Steri-Strip, 3 M Health Care, St Paul, MN, USA)	5 mm (randomized to same patient)	Complications	Wound infection (confirmed by physician); Wound redness (persisting for ≥ 90 days); Wound dehiscence
							Cosmesis	Patient VAS at 90 days; Blinded investigator HWES at 90 days
							Pain	Presence of pain at 90 days
Chen (2010)	Sample (% male): 40 (0%); Age (range): 20–68; Mean BMI (range): 22 (19–25)	Gynecological	Subcuticular suture 4–0 poliglecaprone	Tissue adhesive; 2- octyl cyanoacrylate	NA	5 mm (randomized to same patient)	Complications	Erythema, warmth, drainage, infection
							Cosmesis	Blinded patient HWES at 14–28 days; Blinded clinician HWES at 14–28 days
Dowson (2006)	Sample (% male): 154 (34%); Median Age (range): 48 (20–84); BMI: NR	General Surgical (cholecystectomy, inguinal hernia repair, splenectomy, fundoplication)	Transcutaneous suture 3–0 nylon	Tissue adhesive n-butyl-cyanoacrylate	NA	Median (range) 7.7 mm (5-30 mm) (randomized to different patients)	Complications	Infection defined as ≥ 3 of: erythema, edema, inflammation, drainage, malodor. Wound dehiscence
							Cosmesis	Blinded investigator HWES at 4–6 weeks and 90 days
							Time	From wound closure device pick up to being put down after closure of all wounds
Harold (2004)	Sample (% male): 48 (NR); Mean Age: 48; BMI: NR	General Surgical (cholecystectomy, fundoplication, Heller myotomy, appendicectomy, splenectomy, diagnostic laparoscopy, adrenalectomy, inguinal hernia repair	Subcuticular suture 4–0 polyglactin	Tissue adhesive 2-octyl cyanoacrylate	Adhesive papertape (Steri-Strip Elastic with Compound Benzoin Tincture, 3 M Health Care, St Paul, MN, USA)	5 mm (randomized to individual wounds)	Complications	Wound infection
							Cosmesis	Patient VAS at 1 and 6 weeks; Blinded investigator HWES by photograph at 1 and 6 weeks
							Pain	VAS at 1 and 6 weeks
Jan (2013)	Sample (% male): 114 (0%); Mean Age (SD): 44 (10); Median BMI: 24.5	Gynecological	Subcuticular suture 3–0 polyglactin	Tissue adhesive butyl and octyl cyanoacrylate blend	NA	5 mm (randomized to different patients)	Complications	Erythema, edema, pain, inflammation discharge, odor, dehiscence at 2 weeks
							Cosmesis	Unblinded investigator HWES at 14 days
Jallali (2004)	Sample (% male): 25 (12%); Median Age: adhesive group 36; suture group 56; BMI: NR	Laparoscopic cholecystectomy	Subcuticular suture 3–0 polyglactin	Tissue adhesive 2-octyl cyanoacrylate	NA	5 mm and 10 mm (randomized to different patients)	Complications	ND
							Cosmesis	Unblinded investigator HWES at 6–8 weeks; Blinded plastic surgeon VAS by photograph at 6–8 weeks
							Time	Time to close all wounds by a single method. (including dressing application in the suture group, no dressing required in adhesive group)
Maartense (2002)	Sample (% male): 140 (51%); Mean Age (SEM): 58.7 (2); Mean BMI (SEM): 25.7 (0.9)	General Surgical (cholecystectomy, inguinal hernia repair, fundoplication, diagnostic laparoscopy)	Subcuticular suture poliglecaprone	Tissue adhesive 2-octyl cyanoacrylate	Adhesive papertape (Steri-Strips, Bioplasty/Uroplasty, Geleen, The Netherlands)	5 mm and 10 mm (randomized to different patients)	Complications	Wound infection with spontaneous drainage or requiring drainage of purulent fluid
							Cosmesis	Patient VAS at 10–14 days and 3 months; Blinded clinician VAS and HWES at 10–14 days and 3 months
							Time	From the time the surgeon was ready to start wound closure to completion of closure
Romero (2011)	Sample (% male): 49 (59%); Mean Age (Range): 11 (5–15); Mean BMI (SD): 18.8 (3.1)	Laparoscopic appendicectomy	Adhesive papertape (Steri-Strip, 3 M Medica, Neuss Germany)	Tissue adhesive 2-octyl cyanoacrylate	NA	5 mm and 10 mm (randomized to different patients)	Complications	Wound infection (abscess or erythema > 3 mm perpendicular to incision); Wound dehiscence
							Cosmesis	Patient satisfaction as yes/no at 10 and 90 days; Blinded surgeon VAS at 90 days by photograph
							Pain	Presence of pain at 90 days
Rosen (1997)	Sample (% male): 54 (0%); Mean Age: NR; BMI: NR	Gynecological	Transcutaneous 3–0 Nylon	Subcuticular 3–0 polyglactin	Adhesive papertape	5 and 10 mm (randomized to the same patient)	Complications	Inflammation, discharge, wound dehiscence
							Cosmesis	Patient 5-point scale satisfaction for cosmesis at 4 weeks; Consultant surgeon—poor, satisfactory, perfect
							Pain	5-point pain scale at 5 days
Sebesta (2003)	Sample (% male): 59 (100%); Mean Age: NR; Mean BMI (SD): 31.31 (6.56)	Urological	Subcuticular suture 5–0 polyglactin or poliglecaprone	Tissue adhesives 2- octyl cyanoacrylate	NA	Mean (SD) total length of incisions: 44.16 mm (2.6 mm) (randomized to different patients)	Complications	Infection, dehiscence, seroma

HWES Hollander wound elevation scale, ND not defined, NR not reported, POSAS patient and observer scar assessment scale, SD standard deviation, SEM standard error of the mean, VAS visual analog scale

Harold et al. rated “tissue adhesive closures 4.17 times more likely to have pain (*p *= 0.05)” compared to subcuticular suture and adhesive papertape at 6 weeks [22]. The direct relationship between suture and tissue adhesive was not analyzed. The original data had been destroyed when the authors were contacted for further clarification.

#### Time

All six studies found significantly decreased time taken to achieve wound closure for tissue adhesive compared with suture [14–18, 22]. Table 2 summarizes the time taken to achieve wound closure with suture compared to tissue adhesive.

**Table 2:** Summary of findings and quality of evidence assessment (GRADE)

Outcomes (Author, year)	Summary of findings		Quality of evidence assessment (GRADE)			
	Sample size (no of trials)	Effect size RR^a^ (95% CI^b^)	Study limitation	Inconsistency	Imprecision	Quality
*Transcutaneous suture vs subcuticular suture*						
Infection (Buchweitz, 2005)	104 (1 RCT^c^)	0.25 (0.03–2.16)	No limitation	Inconsistency	Imprecision	Low
Dehiscence (Buchweitz, 2005)	104 (1 RCT)	0.2 (0.01–4.07)	No limitation	Inconsistency	Imprecision	Low
Erythema (Buchweitz, 2005)	104 (1 RCT)	0.25 (0.03–2.16)	No limitation	Inconsistency	Imprecision	Low
Pain (Buchweitz, 2005)	104 (1 RCT)	0.25 (0.03–2.16)	No limitation	Inconsistency	Imprecision	Low
*Transcutaneous suture vs tissue adhesive*						
Infection (Buchweitz, 2016; Dowson, 2006)	231 (2 RCTs)	1.23 (0.48–3.19)	Limitation	Inconsistency	Imprecision	Very Low
Dehiscence (Buchweitz, 2016; Dowson, 2006; Jan, 2013)	576 (3 RCTs)	0.67 (0.17–2.53)	Limitation	No inconsistency	Imprecision	Low
Erythema (Dowson, 2006; Jan, 2013)	464 (2 RCTs)	1.18 (0.77–1.79)	Limitation	No inconsistency	Imprecision	Low
Drainage (Dowson, 2006; Jan, 2013)	464 (2 RCTs)	0.64 (0.14–3.00)	Limitation	No inconsistency	Imprecision	Low
Pain (Dowson, 2006; Jan, 2013)	464 (2 RCTs)	1.18 (0.49–2.85)	Limitation	No inconsistency	Imprecision	Low
Cosmesis HWES at 90 days (Buchweitz, 2016)	112 (1 RCT)	1.02 (0.97–1.07)	No limitation	Inconsistency	Imprecision	Low
Cosmesis HWES at 14 days (Jan, 2013)	345 (1 RCT)	1.01 (0.99–1.03)	Limitation	No inconsistency	Imprecision	Low
*Subcuticular suture vs tissue adhesive*						
Infection (Maartense, 2002; Sebesta, 2003)	316 (2 RCTs)	0.52 (0.15–1.84)	No limitation	No inconsistency	Imprecision	Moderate
Dehiscence (Ben Safta 2020; Sebesta, 2003)	288 (2 RCTs)	0.08 (0.01–0.60)	No limitation	Inconsistency	Imprecision	Low
Erythema (Chen, 2010; Ben Safta, 2020)	150 (2 RCTs)	0.08 (0.01–0.38)	No limitation	Inconsistency	Imprecision	Low
Drainage (Chen, 2010; Sebesta, 2003)	298 (2 RCTs)	3.08 (0.39–24.25)	No limitation	No inconsistency	Imprecision	Moderate
Pain (Chen, 2010)	80 (1 RCT)	16 (2.23–114.98)	No limitation	Inconsistency	Imprecision	Low
Patient dissatisfaction (Ben Safta, 2020)	70 (1 RCT)	0.80 (0.23–2.73)	Limitation	Inconsistency	Imprecision	Very low
Cosmesis HWES at 28 days (Ben Safta, 2020)	70 (1 RCT)	1.15 (0.91–1.46)	Limitation	Inconsistency	Imprecision	Very Low
Cosmesis HWES at 42–56 days (Jallali, 2004)	100 (1 RCT)	1.07 (0.79–1.45)	No limitation	Inconsistency	Imprecision	Low
Cosmesis HWES at 90 days (Maartense, 2002)	98 (1 RCT)	1.27 (1.08–1.49)	No limitation	Inconsistency	Imprecision	Low
*Subcuticular suture vs adhesive papertape*						
Infection (Maartense, 2002; Buchweitz, 2005)	167 (2 RCTs)	1.09 (0.31–3.80)	Limitation	No inconsistency	Imprecision	Low
Erythema (Buchweitz, 2005)	75 (1 RCT)	4.08 (0.23–72.72)	No limitation	Inconsistency	Imprecision	Low
Pain (Buchweitz, 2005)	75 (1 RCT)	4.08 (0.23–72.72)	No limitation	Inconsistency	Imprecision	Low
Cosmesis HWES at 90 days (Maartense, 2002)	98 (1 RCT)	1.47 (1.18–1.83)	No limitation	Inconsistency	Imprecision	Low
*Transcutaneous suture vs adhesive papertape*						
Infection (Buchweitz, 2005)	75 (1 RCT)	0.44 (0.03–6.77)	No limitation	Inconsistency	Imprecision	Low
Erythema (Buchweitz, 2005)	75 (1 RCT)	1.36 (0.06–32.15)	No limitation	Inconsistency	Imprecision	Low
Pain (Buchweitz, 2005)	75 (1 RCT)	1.36 (0.06–32.15)	No limitation	Inconsistency	Imprecision	Low
*Tissue adhesive vs adhesive papertape*						
Infection (Maartense, 2002); Romero, 2011)	137 (2 RCTs)	1.16 (0.33–4.08)	No limitation	No Inconsistency	Imprecision	Moderate
Pain (Romero, 2011)	42 (1 RCT)	1.82 (1.18–18.55)	No limitation	Inconsistency	Imprecision	Low
Cosmesis HWES at 90 days (Maartense, 2002)	98 (1 RCT)	1.16 (0.89–1.51)	No limitation	Inconsistency	Imprecision	Low
Patient dissatisfaction at 10 days (Romero, 2011)	42 (1 RCT)	2.61 (0.56–12.13)	No limitation	Inconsistency	Imprecision	Low
Patient dissatisfaction at 90 days (Romero, 2011)	42 (1 RCT)	2.65 (0.31–24.14)	No limitation	Inconsistency	Imprecision	Low

Grading of recommendations, assessment, development and evaluations

^a^ = Relative risk; ^b^Confidence Intervals; ^c^Randomized controlled trials

#### Cost

Sebesta & Bishoff reported that the average cost per closure with tissue adhesive was $198 vs $497 for suture (*p *< 0.0001) [14]. Maartense et al. found closure with suture to be cheaper than tissue adhesive (*p *< 0.001) [15]. However, operational costs were reported significantly cheaper than rates reported by other studies [15].

### Summary comparison 1: suture vs tissue adhesive

Comparing suture vs tissue adhesive, no difference was identified in postoperative wound complications for infection, dehiscence nor prolonged drainage. One study [21] reported prolonged erythema in wounds closed by subcuticular suture compared with tissue adhesive; however, this study did not report any cases of wound infection.

Superior long-term cosmesis at 90 days was reported in one study with suture closure [15]. These findings were not reproduceable by Jallali et al. who found no difference [17], nor Chen et al. who found tissue adhesive to be superior [21]. However Chen et al. assessed cosmesis at 2 weeks postoperatively [21]. This timeframe may be too soon to adequately to assess cosmesis as wound healing and scar maturation only commences at this point.

Decreased surgeon satisfaction reported by Maartense et al. in achieving wound closure by tissue adhesive may have been confounded by a surgeon learning curve [15]. There was no difference in patient satisfaction or prolonged presence of pain. Tissue adhesive closure is significantly faster than suture, halving closure time, with resultant cost savings despite the increased cost of a single unit of tissue adhesive compared with suture [15].

### Comparison 2: suture vs adhesive papertape

#### Primary outcome: wound complications

In comparing transcutaneous sutures with adhesive papertape, one study found no statistically significant difference (RR: 0.44, 95% CI: 0.03–6.77) (Fig. 3) [20]. In comparing subcuticular sutures with adhesive papertape for infection, one study reported no cases of wound infection and was therefore not included in meta-analysis [22]. Pooled data from the two remaining studies [15, 20] showed no difference (RR: 1.09, 95% CI: 0.31–3.80) (Fig. 3).

**Fig. 5:**
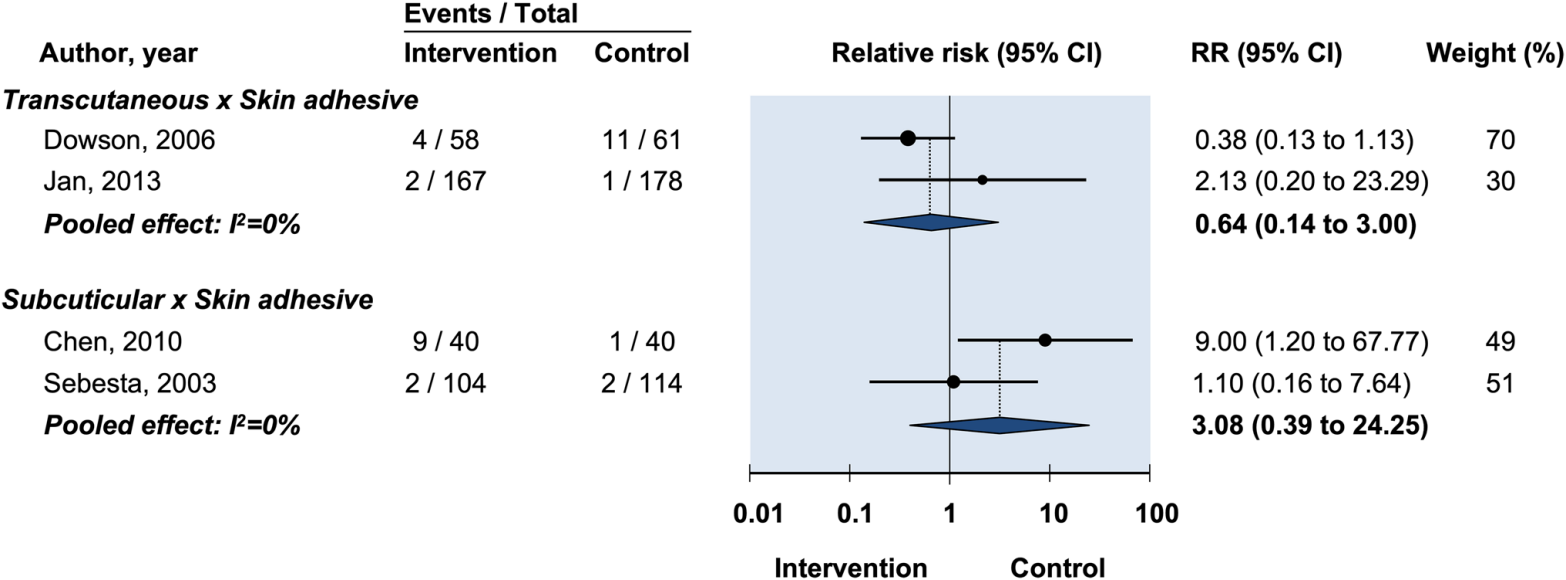
Relative risk of postoperative wound drainage in randomized controlled trials on efficacy of optimal wound closure of abdominal laparoscopic port sites. Relative risk (RR) < 1 favor intervention group. CI = Confidence intervals

One study made comparisons between both transcutaneous and subcuticular sutures against adhesive papertape for dehiscence (RR: 0.88, 95% CI: 0.08–9.27 and RR: 0.15, 95% CI: 0.006–3.57 respectively) (Fig. 4). No statistically significant difference was found [20] (Fig. 5).

**Fig. 6:**
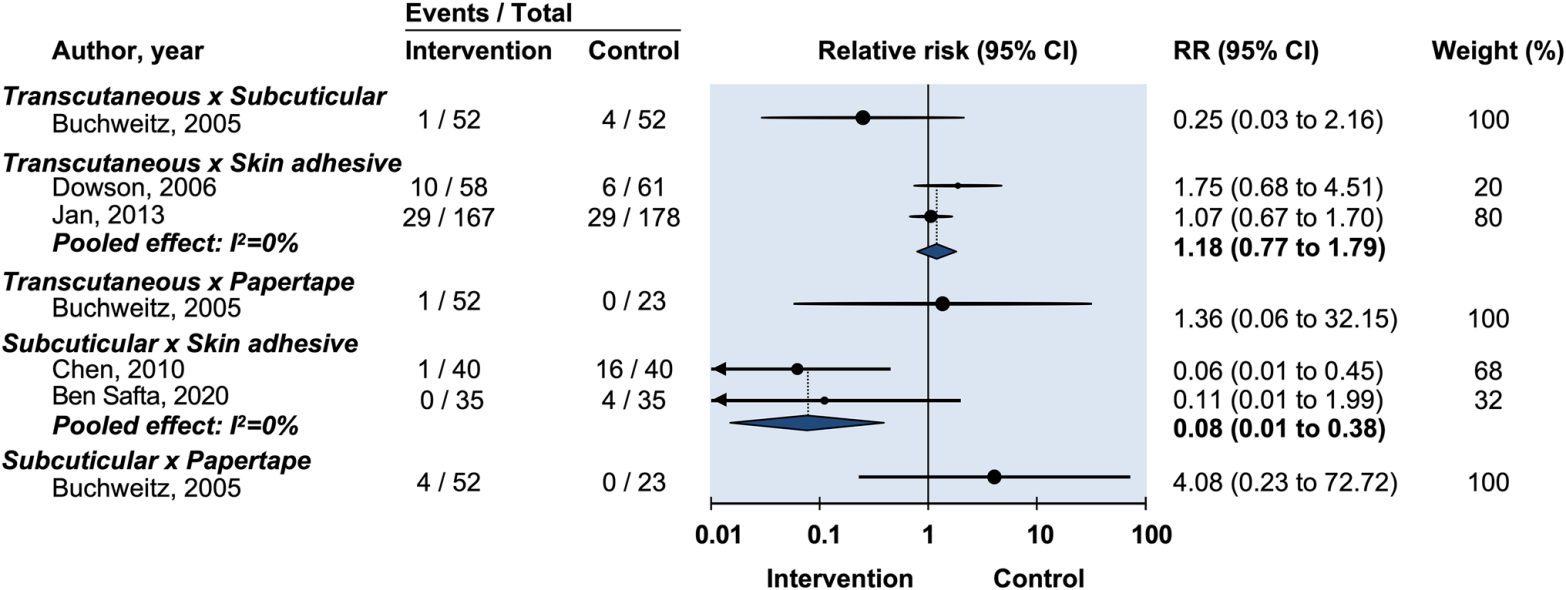
Relative risk for postoperative erythema in randomized controlled trials on efficacy of optimal wound closure of abdominal laparoscopic port sites. Relative risk (RR) < 1 favor intervention group. CI = Confidence intervals

One study reported on persistent erythema. There was no difference found in either transcutaneous or subcuticular sutures when compared with adhesive papertape (Fig. 6) [20].

**Fig. 7:**
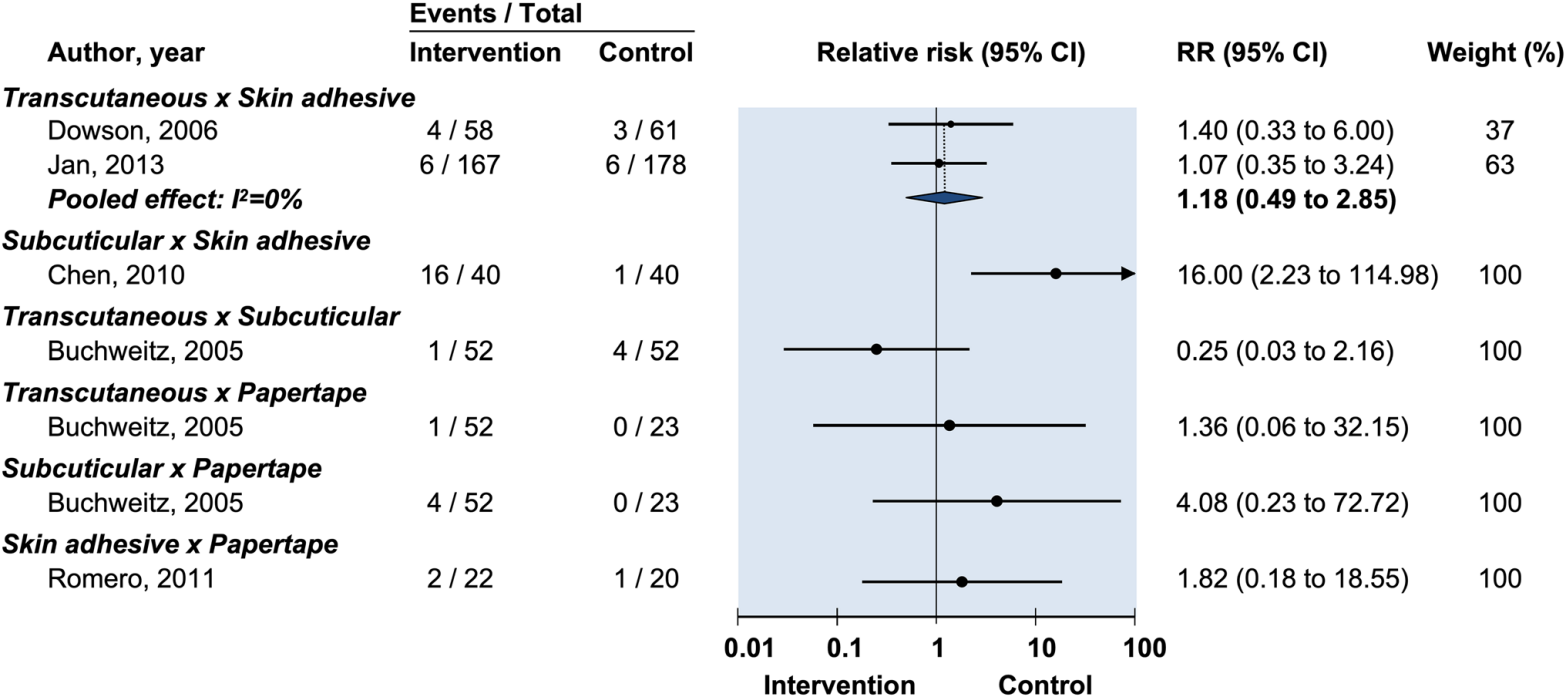
Relative risk of postoperative pain in randomized controlled trials on efficacy of optimal wound closure of abdominal laparoscopic port sites. Relative risk (RR) < 1 favor intervention group. CI = Confidence intervals

One study grouped all complications together and was not pooled. The authors were contacted for data; however, no reply was received. No statistically significant difference in overall complication rate was identified when comparing transcutaneous and subcuticular sutures against adhesive papertape [23] (Fig. 7).

### Secondary outcomes

#### Overall patient satisfaction

**Fig. 8:**
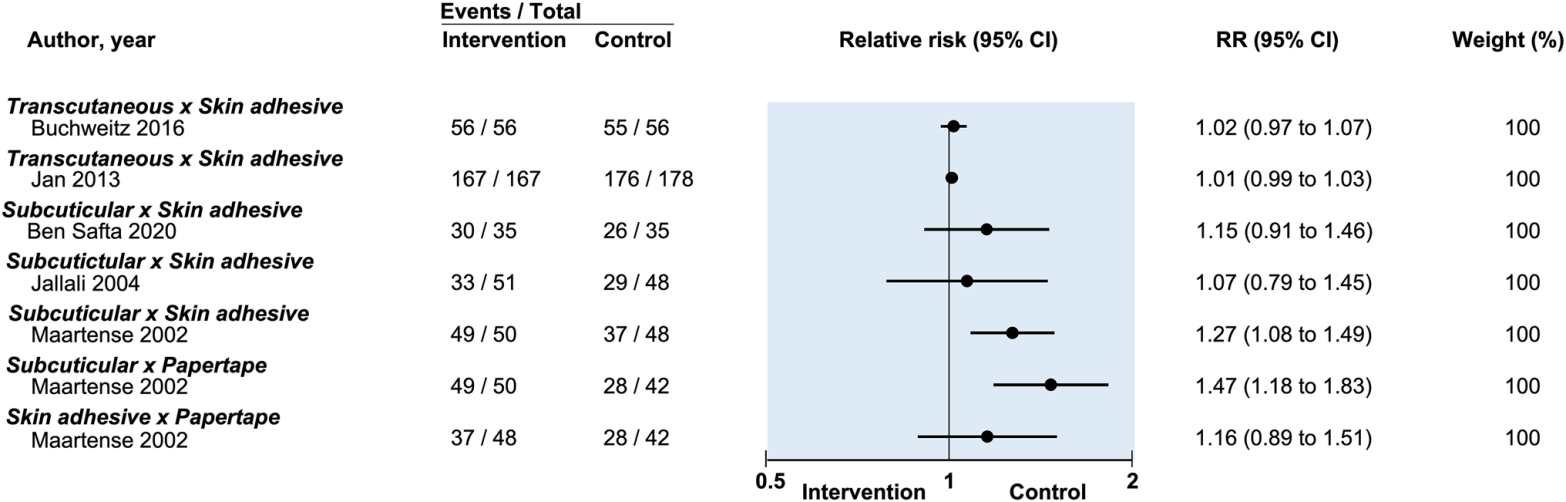
Relative risk for postoperative HWES (Hollander Wound Evaluation Score) in randomized controlled trials on efficacy of optimal wound closure of abdominal laparoscopic port sites. Relative risk (RR) < 1 favor intervention group. CI = Confidence intervals

One study reported that no statistically significant difference was found in overall patient satisfaction between transcutaneous, subcuticular suture and adhesive papertape closure [23] (Fig. 8).

#### Surgeon satisfaction

One study reported on surgeon rating of practicality. Adhesive papertape was rated as not practical in 14.3% vs 0% for suture closure, *p *= 0.0059 [15].

#### Cosmesis

One study evaluated cosmesis with HWES at 90-day follow-up and found significantly higher rates of perfect HWES score for subcuticular suture (97%) compared with adhesive papertape (42%) (RR: 1.47, 95% CI: 1.18–1.83) [15].

Two studies reported patient-assessed VAS scores. Pooled data showed no difference in cosmesis between the two groups (MD: −0.16, 95% CI: −0.68 to 0.36) (Fig. 4) [15, 20].

**Fig. 9:**
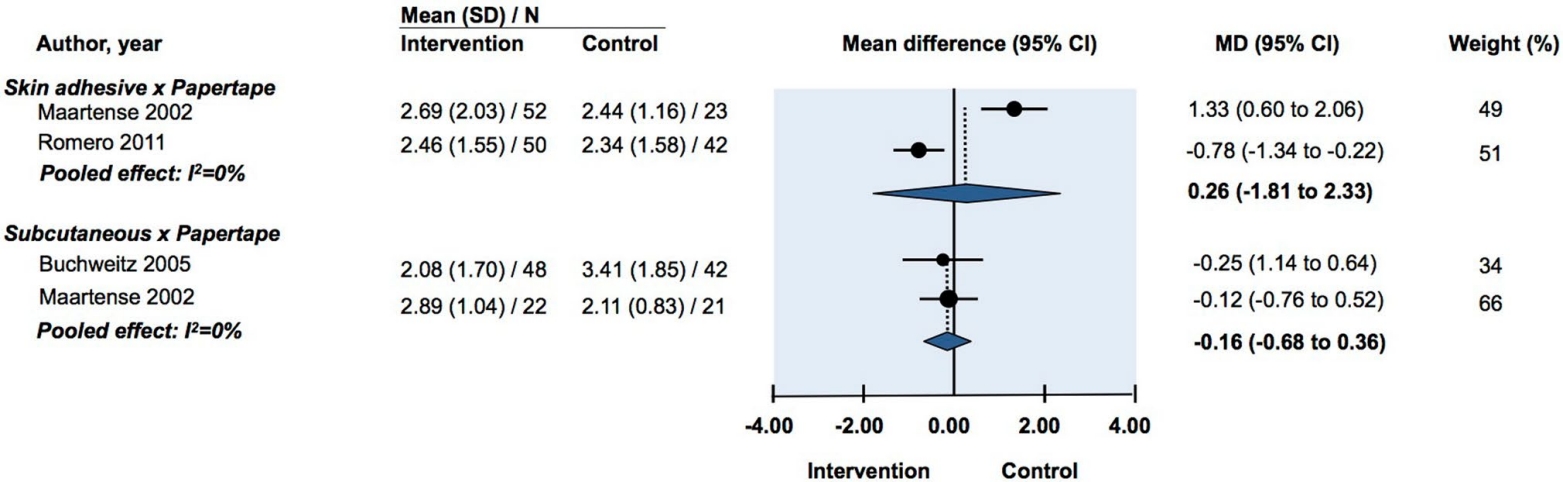
Mean difference for cosmesis as assessed by VAS at 90 days in randomized controlled trials investigating the efficacy of optimal wound closure of abdominal laparoscopic port sites. Negative values favor intervention. MD = Mean difference. CI = Confidence intervals

One study reported no significant difference in overall blinded observer satisfaction between transcutaneous suture, subcuticular suture and adhesive papertape closure in 5 mm and 10 mm port wounds. There was however a statistically significantly lower degree of satisfaction among blinded observers assessing transcutaneous umbilical wound closure against subcuticular and adhesive papertape closure when assessed 4 weeks postoperative (*p *< 0.05) [23] (Fig. 9).

#### Pain

One study reported the percentage of painful scars at 90 days to be 7.7% in subcuticular closure of wounds, 1.9% in transcutaneous closure wounds and 0% in adhesive papertape; however, this was not statistically significant [20]. Harold et al. reported “Cyano-acrylate tissue adhesive closures 4.17 times more likely to have pain (*p *= 0.05)” compared to suture and adhesive papertape at 6 weeks [22]. However, no numerical data was provided other than graphical representation with approximated percentages at 8% for suture vs 3% for adhesive papertape [22]. When the authors were contacted for further data, their original data had been disposed from available records and no further analysis was possible.

One study reported the least amount of pain in subcuticular sutures compared to transcutaneous sutures and adhesive papertape (*p *< 0.05) [23]. No numerical values were provided by the authors and no reply was received when further information was requested.

#### Time

One study reported time taken to close per patient to be significantly less for adhesive papertape compared with suture, median 228 (range 65–894) seconds vs 80 (20–571) seconds, *p *< 0.001 [15]. Another study reported no difference between the two groups [22]; however, this study provided no information on definition of time taken to close and how this was data was collected.

#### Cost

Cost differences were reported by Maartense et al. who reported cost of closure per patient to be $18.29 (3.59–68.69) for suture and $9.40 (2.86–47.19) for adhesive papertape (*p *> 0.001) [15].

### Summary: suture vs adhesive papertape

In comparing suture vs adhesive papertape, there was no difference in wound complications nor patient-assessed cosmesis at 90 days. Investigator assessed cosmesis was better for subcuticular sutures compared with adhesive papertape. However, the clinical application is debatable given patient satisfaction was not significantly different. Closure of umbilical skin with transcutaneous sutures was rated less cosmetically satisfying than adhesive papertape. Adhesive papertape was faster to apply with an associated cost saving but more frequently reported as impractical by surgeons.

### Comparison 3. tissue adhesive vs adhesive papertape

#### Primary outcome: wound complications

One study reported no cases of infection, leaving two studies for inclusion in meta-analysis [12, 15, 22]. Pooled analysis found no significant difference between the two groups (RR: 1.16, 95% CI: 0.33–4.08) (Fig. 3) [12, 15].

### Secondary outcomes:

#### Overall patient satisfaction

Not investigated.

#### Surgeon satisfaction

One study rated surgeon practicality. Adhesive papertape was rated ‘not practical’ in 14.3% vs 6.3% in tissue adhesive, but this was not statistically significant (RR:0.52, 95% CI:0.14–1.97) [15].

#### Cosmesis

Pooled analysis of two studies reporting blinded investigator VAS score for cosmesis found no difference between the two groups (MD: 0.26, 95% CI: −1.81 to 2.33) (Fig. 4) [12, 15, 22]. Another study reported investigator-rated HWES and patient-rated VAS. There was no difference between the groups in either outcome measurement for cosmesis [15].

#### Pain

Romero et al. found no difference in pain at 90-day follow-up (RR: 1.82, 95% CI: 0.18–18.55) [12]. Harold et al. rated “tissue adhesive closures 4.17 times more likely to have pain (*p *= 0.05)” compared to suture and adhesive papertape at 6 weeks [22]. The direct relationship between tissue adhesive and adhesive papertape was not analyzed. The original data had been destroyed when the authors were contacted for further clarification.

#### Time

Two studies reported a slightly faster closure time with adhesive papertape than tissue adhesive [15, 22]. Harold et al. records a difference of mere seconds 34.7 ± 24.5 vs 33.4 ± 20.8 s (not statistically analyzed). Maartense et al. found no significant difference in time to close wounds with adhesive papertape (median 33 s (7–140)) compared with tissue adhesive (26 s (7–143)). Cumulative time taken for each patient was 119 (22–420) seconds for tissue adhesive and 80 (20–571) seconds for adhesive papertape (*p *< 0.05) [15].

#### Cost

Maartense et al. reported cost of closure per patient to be $36.82 (16.60–67.11) for tissue adhesive and $8.68 (2.86–47.20) for adhesive papertape (*p *> 0.001) [15].

### Summary: tissue adhesive vs adhesive papertape

In comparing tissue adhesive with adhesive papertape, there was no difference in wound complications. Surgeons reported adhesive papertape to be ‘not practical’ more frequently than for tissue adhesive. No superiority in cosmesis as reviewed by both investigators and patients was demonstrated for either group. Closure is slightly faster with adhesive papertape although clinically, this small difference is unlikely to account for any practical difference. Adhesive papertape was significantly cheaper than tissue adhesive.

### Comparison 4: subcuticular vs transcutaneous suture

#### Primary outcome: wound complications

Two studies demonstrate that the total number of wound complications (infection, dehiscence, persistent erythema) more commonly complicates subcuticular closure compared with transcutaneous closure (17.3% vs 3.8%) (RR: 0.22, 95% CI: 0.05–0.98). There was no statistical significance when analysis was broken down into infection, dehiscence and persistent erythema [20, 23].

### Secondary outcome

#### Overall patient satisfaction

One study studied this with no statistically significant difference in overall patient satisfaction measured with a 5-point scale [23].

#### Surgeon satisfaction

Not evaluated.

#### Cosmesis

One study reported significantly more dissatisfying cosmetic results after subcuticular sutures compared with transcutaneous sutures (*p *= 0.039). Patient assessment with VAS (best score of 0) was 1.9 for transcutaneous compared with 2.44 for subcuticular closure (*p *= 0.005) [20]. Another study reports transcutaneous closure for the umbilicus statistically significantly rated poorer by blinded observers, however there was no difference in closure between transcutaneous and subcuticular sutures elsewhere [23].

#### Pain

One study reported no statistically significant difference in prolonged pain between subcuticular and transcutaneous sutures (RR: 0.25, 95% CI: 0.03–2.16) (Fig. 4) [20].

**Fig. 10:**
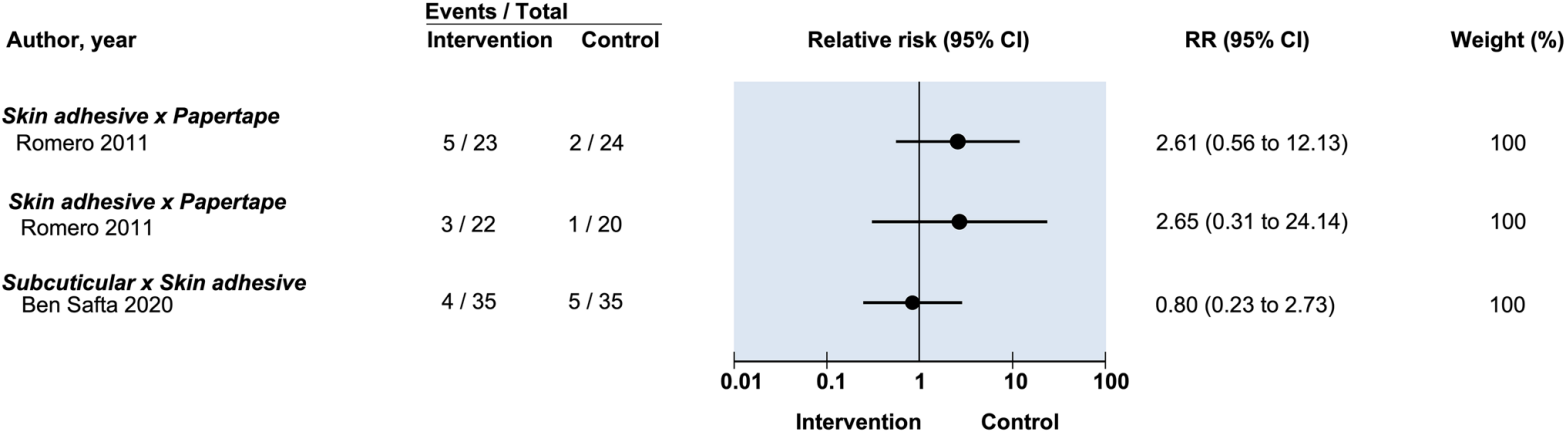
Relative risk of postoperative dissatisfaction in randomized controlled trials on efficacy of optimal wound closure of abdominal laparoscopic port sites. Relative risk (RR) < 1 favor intervention group. CI = Confidence intervals

Rosen & Carlton reported less pain in subcuticular sutures compared to transcutaneous sutures (*p *< 0.05) [23]. No numerical values were provided by the authors and no reply was received when further information was requested (Fig. 10).

#### Time

Not evaluated.

#### Cost

Note evaluated.

### Summary: subcuticular vs transcutaneous suture

Overall, the results favor transcutaneous suture closure over subcuticular closure with statistically significant decreased rate of wound complications and improved cosmesis. Patient satisfaction is not altered by choice of suture closure. Patient rated cosmesis is better for wounds closed with transcutaneous sutures compared with subcuticular sutures. Blinded observers rated transcutaneous closure of umbilical wounds to have worse cosmesis than subcuticular sutures to the same location. Pain from subcuticular sutures may be less than transcutaneous sutures; however, this was not reproducible.

## Discussion

### Statement of principal findings

The results of this meta-analysis indicate that there was no evidence of difference in postoperative wound complications (infection, dehiscence, prolonged drainage and prolonged erythema) for suture vs tissue adhesive, for suture vs adhesive papertape or for tissue adhesive vs adhesive papertape. Transcutaneous suture has a decreased rate of overall wound complications when compared with subcuticular sutures.

For overall patient satisfaction, there was no difference in comparison of suture vs tissue adhesive nor suture vs adhesive papertape. Surgeons found closure with tissue adhesive and adhesive papertape to both be less practical than suture.

For patient-rated cosmesis, there was conflicting and non-reproducible evidence comparing suture and tissue adhesive, and suture and adhesive papertape. There was no evidence demonstrating a higher rating when comparing adhesive papertape and tissue adhesive.

It is uncertain as to whether there are differences in persistence of pain between the closure methods. Regardless, overall numbers of prolonged pain in all groups were minimal.

Wound closure by tissue adhesive, and adhesive papertape were both significantly faster than suture, with a 1.5–3.5 min operative time reduction per patient. One study reported a time saving of ten minutes for tissue adhesive compared with suture; however, this is likely resultant from their median wound length being longer than other studies. Adhesive papertape was faster than tissue adhesive although this statistical significance did not translate to clinical significance as the absolute time saving was less than one minute. Given the time-savings, closure by tissue adhesive and adhesive papertape were significantly cheaper compared with suture. Adhesive papertape was overall cheaper than tissue adhesive.

Many of the trials included in the study were small and, combined with individual trial quality resulted in evidence being only moderate, low or very low. Sources of trial weakness include issues with unit analysis, “some concerns” or “high” risk of bias, and heterogeneity between trial definitions of measured outcomes. Additionally, several patient factors (for example BMI (Body Mass Index), diabetes, smoking), as well as surgical factors (clean vs contaminated) that are known to impact wound healing were not controlled for within the individual studies.

In particular, BMI may play a particularly important role in surgical decision-making for choice of skin closure method. Possible increased tension on the incisions of obese patients may theoretically warrant a closure method that has more tensile strength. A study comparing tensile strength of 2-Octyl cyanoacrylate tissue adhesive with staples, adhesive papertape and interrupted 4–0 poliglecaprone subcuticular sutures found that staples were able to withstand the highest tensile strength and that tissue adhesive was superior to adhesive papertape. Tissue adhesive was comparable to suture [25]. The role of BMI in choice of skin closure material thus requires further investigation.

Some outcome data were presented by wound rather than by patient. This may introduce a source of clustering that was not accounted for. On the other hand, other studies were presented by patient rather than by wound. Some of these studies failed to report how many of the wounds in a single patient were affected and thus may have under-reported the number of wound complications.

Almost all studies provided no criteria for definition of wound infection, thus presenting the possibility of significant heterogeneity.

This meta-analysis is limited by the number of RCTs available in the literature that focus in on the same interventions and outcomes that allowed for meaningful multivariate analyses.

Furthermore, it is difficult to determine if confounding factors may have impacted the outcomes of the individual RCTs. Many did not control for these. Future publications should consider individual patient data meta-analysis where these variables could be adjusted for.

Another potential source of error present in some of the included studies was that they relied upon patient questionnaires to report wound complications. A systematic review evaluating methods for identifying surgical wound infection following hospital discharge found that patient self-diagnosis was unreliable with high false-positive and false-negative rates. Whitby et al. [26] compared post-discharge patient-reported complications (via questionnaire) against infection-control nurse and physician assessment and found that patients only had a 29% positive predictive value.

Tissue adhesive was reported to be less practical than suture closure. However, given that suturing is currently more commonly practised, studies may fail to take into account the learning curve associated with tissue adhesive (particularly in "surgeon satisfaction") and future studies should attempt to eliminate this prior to data collection.

### Comparison with other studies

A Cochrane review published by Dumville et al. compared the use of tissue adhesives with other skin closure techniques for the closure of any surgical incision [7]. They included 33 studies involving 2793 patients from all surgical specialties, with operations ranging from total hip replacements, to episiotomies, to facial lacerations. They found low-quality evidence that sutures were significantly better than tissue adhesives for reducing the risk of wound dehiscence (RR: 3.35, 95% CI: 1.53–7.33 with NNT 43). Adhesive papertape was found to be favored against tissue adhesives in blinded investigator assessment of cosmesis. Tissue adhesives were faster than sutures. However for all other outcomes—infection, patient and surgeon satisfaction there was no difference [7].

The findings of Dumville et al. are consistent with our own despite the significant heterogeneity in the types of wounds they assessed. The main finding that differed from ours was that there was increased risk of dehiscence with tissue adhesive compared with sutures [7]. This difference is likely a result of the increased wound lengths and increased tension of the wounds included in their study.

Our study included one study that compared transcutaneous vs subcuticular sutures and found decreased wound complications and improved cosmesis with the transcutaneous method [20]. A RCT of 100 patients undergoing open heart surgery compared closure of skin in sternotomy wounds with either transcutaneous or subcuticular suture methods and found infection rates to be increased with subcuticular sutures (2% vs 16%, *p *= 0.016) and to have no difference in cosmesis [27]. Another RCT of 70 patients conducted on open appendicectomy wounds found that the width of the scar, and pain at day seven postoperatively were decreased in subcuticular closure, and patient satisfaction was increased in the subcuticular group [28]. No difference was found in wound complications; however, the study was not powered to detect this. While the current low-quality evidence demonstrates that transcutaneous sutures show decreased rates of complications, this should be balanced with the burden on the individual patient and the healthcare system to have sutures removed postoperatively. This may account for the decreased patient satisfaction and increased pain at day seven, demonstrated in the study by Javadi et al. [28].

While Kent, Liversedge & Dobbins was excluded from our study, they provide useful comparison between octyl-butyl cyanoacrylate blend vs octyl cyanoacrylate in the closure of laparoscopic port sites. In their randomized, double-blinded study of 433 patients they found no difference in complication rate, which was low for both groups [29]. There was no difference in satisfaction with wound appearance; however, surgeons found the octyl-butyl cyanoacrylate blend to be significantly easier to use with higher surgeon satisfaction [29].

## Conclusion

Given the rates of wound complication following laparoscopic surgery are low, adequately powered, large randomized trials are needed to detect a statistically significant difference between closure methods using a standardized definition for superficial surgical site infection and wound dehiscence.

The results from 12 randomized controlled trials across multiple surgical specialties found no evidence that there was increased wound complications with suture, tissue adhesive or adhesive papertape closure of laparoscopic port sites.

There was no evidence of a difference in cosmesis, prolonged pain, or patient satisfaction between the three groups. Low-quality evidence showed that transcutaneous sutures may have reduced wound complications and marginally improved cosmesis compared with subcuticular sutures, but this needs to be weighed against the need for additional follow-up for suture removal.

Tissue adhesive and adhesive papertape offer safe, cost, and time-saving alternatives to suture closure of laparoscopic port sites.

## Supplementary Information

Below is the link to the electronic supplementary material.

Supplementary file1 (DOCX 14 KB)
