# Supplementary material for: To stitch or not to stitch: the skin closure of laparoscopic port sites, a meta-analysis
Source: Surg Endosc. 2022 May 24;36(10):7140–59. doi: 10.1007/s00464-022-09269-9 (PMC9485090; doi:10.1007/s00464-022-09269-9)
Supplement: Supplementary file 1 — Supplementary file1 (DOCX 14 KB) [file 464_2022_9269_MOESM1_ESM.docx]

**Appendix:**

**Supplementary Table 1. Searches strategies for each database**

The following medical subject headings and keywords were used to identify potential articles to search Ovid MEDLINE.

1. Laparoscop*
2. Abdominoscop*
3. Peritoneoscop*
4. Celioscop*
5. Coelioscop*
6. Laparoscop* surgery
7. Minimally invasive surgery
8. #1 OR #2 OR #3 OR #4 OR #5 OR #6 OR #7
9. Sutur*
10. Suture technique*
11. Stitch*
12. Liquid stitch*
13. Tape*
14. Glue*
15. Tissue adhesive*
16. Acrylate*
17. Cyanoacrylate*
18. Octylcynoacrylate*
19. Bucrylate*
20. Stapl*
21. Surgical-stapling
22. #9 OR #10 OR #11 OR #12 OR #13 OR #14 OR #15 OR #16 OR #17 OR #18 OR #19 OR #20 OR #21
23. #8 AND #22
24. Randomized controlled trial OR Randomised controlled trial OR Controlled clinical trial OR clinical trial OR randomly OR trial OR random*
25. #23 AND #24

| Pubmed | Search ((((((laparoscop* OR laparoscopic surgery OR keyhole OR celioscop* OR coelioscop* OR abdominoscop* OR peritoneoscop*)) AND (port OR port site OR keyhole)) AND (close OR closure OR skin OR paper tape OR paper OR adhesive OR suture)) AND ("1998/01/01"(PDAT) : "2018/12/31"(PDAT)) AND Humans(Mesh))) AND ((randomized controlled trial(Publication Type) OR (randomized(Title/Abstract) AND controlled(Title/Abstract) AND trial(Title/Abstract)))) Sort by: Best Match Filters: Publication date from 1998/01/01 to 2019/01/20; Humans |
| --- | --- |
| Embase | #1 (Randomised or control or controlled clinical trial or trial).mp. (mp=title, abstract, heading word, drug trade name, original title, device manufacturer, drug manufacturer, device trade name, keyword, floating subheading word, candidate term word)  #2 (Port or laparoscopic port).mp. (mp=title, abstract, heading word, drug trade name, original title, device manufacturer, drug manufacturer, device trade name, keyword, floating subheading word, candidate term word)  #3 (Wound or Surgical wound or wound healing).mp. (mp=title, abstract, heading word, drug trade name, original title, device manufacturer, drug manufacturer, device trade name, keyword, floating subheading word, candidate term word)  #4 (Close or closure or tissue adhesive* or suture* or papertape).mp. (mp=title, abstract, heading word, drug trade name, original title, device manufacturer, drug manufacturer, device trade name, keyword, floating subheading word, candidate term word)  #5(laparoscop* or abdominoscop* or peritoneoscop* or celioscop* or coelioscop* or abdominal surgery).mp. (mp=title, abstract, heading word, drug trade name, original title, device manufacturer, drug manufacturer, device trade name, keyword, floating subheading word, candidate term word)  #1AND#2AND#3AND3#4AND#5 |
| CENTRAL | 'Peritoneoscopes; Peritoneoscope; Laparoscope; Celioscope; Celioscopes in Title Abstract Keyword AND wound OR skin OR incision in Title Abstract Keyword AND suture OR adhesive OR tape in Title Abstract Keyword - (Word variations have been searched)' |
